# Zebrafish Lacking Circadian Gene *per2* Exhibit Visual Function Deficiency

**DOI:** 10.3389/fnbeh.2018.00053

**Published:** 2018-03-13

**Authors:** Deng-feng Huang, Ming-yong Wang, Wu Yin, Yu-qian Ma, Han Wang, Tian Xue, Da-long Ren, Bing Hu

**Affiliations:** ^1^Hefei National Laboratory for Physical Sciences at the Microscale and CAS Key Laboratory of Brain Function & Disease, School of Life Sciences, University of Science and Technology of China, Hefei, China; ^2^School of Biology & Basic Medical Sciences, Medical College, Soochow University, Suzhou, China; ^3^Neurodegenerative Disorder Research Center, University of Science and Technology of China, Hefei, China

**Keywords:** *per2*, optokinetic response, contrast sensitivity, ribbon synapses, visual motor response, opsin

## Abstract

The retina has an intrinsic circadian clock, but the importance of this clock for vision is unknown. Zebrafish offer many advantages for studying vertebrate vision and circadian rhythm. Here, we explored the role of zebrafish *per2*, a light-regulated gene, in visual behavior and the underlying mechanisms. We observed that *per2* mutant zebrafish larvae showed decreased contrast sensitivity and visual acuity using optokinetic response (OKR) assays. Using a visual motor response (VMR) assay, we observed normal OFF responses but abnormal ON responses in mutant zebrafish larvae. Immunofluorescence showed that mutants had a normal morphology of cone photoreceptor cells and retinal organization. However, electron microscopy showed that *per2* mutants displayed abnormal and decreased photoreceptor ribbon synapses with arciform density, which resulted in retinal ON pathway defect. We also examined the expression of three cone opsins by quantitative real-time PCR (qRT-PCR), and the expression of long-wave-sensitive opsin (*opn1lw*) and short-wave-sensitive opsin (*opn1sw*) was reduced in mutant zebrafish larvae. qRT-PCR analyses also showed a down-regulation of the clock genes *cry1ba* and *bmal1b* in the adult eye of *per2* mutant zebrafish. This study identified a mechanism by which a clock gene affects visual function and defined important roles of *per2* in retinal information processing.

## Introduction

The circadian clock is an endogenous oscillator that drives daily rhythms in many living systems. In mammals, the master pacemaker is located in the suprachiasmatic nucleus, which generates a regular rhythm with an approximate 24-h period (Reppert and Weaver, 2002; Ko and Takahashi, 2006). The retinal clock was the first extra-SCN circadian oscillator discovered in mammals and is an endogenous circadian clock that regulates many physiological processes within the neural retina (Tosini et al., 2008). A variety of visual behaviors, including photoreceptor cell disk shedding (Besharse, 1982), retinomotor movements (Besharse, 1982; Douglas et al., 1992), and photoreceptor synaptic ribbons (Wagner, 1973), display robust day-night rhythms. The circadian clock is thought to adjust the visual sensitivity to ambient light levels, although the involvement of specific clock genes is poorly understood, particularly in zebrafish.

Based on extensive research, this clock consists of interlocking feedback loops in which the CLOCK-BMAL1 transcription factor drives the expression of *Per* and *Cry* genes (Ko and Takahashi, 2006). According to a previous study, deletion of the core clock component *bmal1* leads to defects in the physiological processing of vision in mice (Storch et al., 2007). Mice lacking *per1* and *per2* show significant alterations in the distribution of cone photoreceptors (Ait-Hmyed et al., 2013). Moreover, in the absence of *Rev*-*erbα*, mouse retinas modify the scotopic threshold responses and increase pupillary constriction, thereby increasing the sensitivity to light (Hakkari et al., 2016). These studies defined the important role of the clock component in eye vision research.

Zebrafish offer many advantages for studying vertebrate vision and circadian rhythmicity, and the zebrafish retina is similar in structure and function to those of other vertebrates, including mammals (Easter and Nicola, 1996). The molecular mechanisms of circadian rhythm generation in zebrafish appear to have much in common with the more extensively studied mammalian system, although the details are different (Cahill, 2002). Zebrafish homologs of three *per* genes have also been identified: two *per1* homologs (*per1a* and *per1b*) and a single copy of the *per2* and *per3* genes. Zebrafish *per2* is a light-regulated gene, and its expression is significantly damped under constant darkness (Vatine et al., 2009). Similar to *cry1a*, *per2* has also been suggested to act as a photoreceptor and is crucial for the *aanat2* rhythms established in the zebrafish pineal gland (Ziv et al., 2005; Tamai et al., 2007). Furthermore, a more recent study of PER2 loss-of-function fish generated by the TALEN method revealed PER2 may serve both as a transcriptional coactivator and a corepressor for Ror/Reverb response element enhancers and E-box enhancers, respectively (Wang et al., 2015).

Currently, little is known about mechanisms underlying how clock gene modulates retinal physiological responses to light. In this study, we used zebrafish as a model to investigate the function of *per2*. We used a variety of anatomical, molecular biological and behavioral approaches to explore the retinal phenotype of zebrafish larvae that lacked the *per2* gene. Data showed that *per2*^−/−^ zebrafish larvae displayed abnormal photoreceptor ribbon synapses and decreased ribbon synapses with arciform density, which led to visual behavior deficiency compared with the wild-type (WT) fish.

## Materials and Methods

### Animals and Maintenance

*Per2* mutant zebrafish were obtained using the TALEN genome-editing tool (Wang et al., 2015). WT AB strain and *per2* mutant zebrafish were maintained and raised on a 14-h/10-h light/dark (LD) cycle at 28°C as previously described (Ren et al., 2015). For dark/dark (DD) experiments, the embryos were removed from the LD cycle at 20:00 h on the day before the experiment and were then kept under DD conditions. All animal manipulations were conducted in strict accordance with the guidelines and regulations set forth by the University of Science and Technology of China (USTC) Animal Resources Center and University Animal Care and Use Committee. The protocol was approved by the Committee on the Ethics of Animal Experiments of the USTC (Permit Number: USTCACUC1103013).

### Optokinetic Response Assays

The optokinetic response (OKR) test was performed as previously described (Mueller and Neuhauss, 2010). Briefly, we used the computer software LabVIEW to generate a sine-wave grating that was projected by an LCD projector (NEC 280+; NEC Corporation, Japan). Zebrafish larvae were immobilized in 6% methylcellulose in a 35-mm petri dish and placed dorsal side up above a small infrared light. With the rotating grating patterns presented around the larva, the elicited eye movement was recorded in real time by an infrared-sensitive CCD camera (TCA-1.3BW; Nanjing, China). WT and *per2* mutant larvae were stimulated with a constant angular velocity of 7.5 degree/s and a fixed spatial frequency (SF) of 0.04 cycle/degree. To measure the visual acuity, the SF was presented with 0.02, 0.04, 0.06 and 0.08 cycle/degree. We used the gain (ratio of eye velocity and stimulus velocity) of the OKR to measure contrast sensitivity (Rinner et al., 2005).

### Visual Motor Response Assay

The visual motor response (VMR) assay was based on a published design (Emran et al., 2007, 2008). The assay was conducted inside a ZebraBox system (ViewPoint Life Sciences, Lyon, France). Mutants and AB-WT were each placed in one of 48 wells of a 96-well plate. Before the actual experiment, the 96-well plate with the larvae was dark-adapted in the ZebraBox system for 3.5 h to acclimatize the animals. The light change (on or off) was abrupt and instantaneous. Larval movement was summarized as the fraction of frames in which a larva displayed movement in each second. The data were processed and analyzed using custom PERL software and Visual Basic Macros for Microsoft Excel.

### RNA Extraction and Quantitative Real-Time PCR (qRT-PCR)

Total RNAs were extracted from approximately 30 larvae of the homozygous *per2* or WT fish at different times (ZT 0 = lights on) of the day and night under LD or DD conditions, and from 3-month adult retinas using RNAiso Plus (Takara) reagent as the manufacturer’s protocol, respectively. Specifically, adult zebrafish retinas were rapidly peeled from the eye tissues with forceps after deeply anesthesia with MS222, and the tissue was frozen at −80°C until further assays. Then, RNAs were reverse transcribed into cDNA using a HiScript^®^ II 1st Strand cDNA Synthesis Kit (Vazyme, Jiangsu, Nanjing, China) according to the manufacturer’s protocol. Gene expression was analyzed by qPCR using SYBR Green Master Mix (Vazyme, Jiangsu, NanJing, China) on a LightCycler^®^ 96 System (Roche Life Science). The PCR thermal profiles were 40 cycles of 10 s at 95°C and 30 s at 60°C. Two housekeeping genes (*β-actin*, *rpl13a*) were assessed for stability in adult retinas; *β-actin* was more stable and was used for all subsequent analyses. All results were normalized to the expression level of the housekeeping gene *β-actin*. The gene-specific primers for *per1b*, *per2*, *per3*, *bmal1b*, and *cry1ba* have been described previously (Wang et al., 2015). Primers for *clock1a*, *cacan1fa*, *synj1*, *opn1lw*, *opn1mw*, *opn1sw* were designed using Primer3 (Rozen and Skaletsky, 2000) and are listed in Table 1. The relative levels of each sample were calculated by the 2^−ΔΔCT^ method (Livak and Schmittgen, 2001).

**Table 1:** Primer used in the study.

Gene	Primer sequence	Accession no
*clock1a*	TTTTGTTGCCACATGCTCCG	NM_130957
	CTAGCCTGACCGTCGCTATG	
*cacna1fa*	CCCCTAGAAGCACGCCTATG	XM_021478483.1
	CCACTTGCTGGGTAAGGGAG	
*synj1*	TACCTGCTCCTCTGATGCCT	NM_001007030.2
	AACCTGAGGATTGCTCCTGC	
*opn1lw*	TGGAGCAGATACTGGCCTCAT	NM_001313715.1
	GGGTCCTCGCTTCCACTGA	
*opn1mw*	GCTGCCACTTTTGCATACCC	NM_131253.2
	TGTCACTTCCCTCTCAGCCT	
*opn1sw*	GTTCGATGGAAGCGGCAATG	NM_131319.1
	ACAGGCGGTACCAATGATCC	

### Immunohistochemistry

Immunohistochemistry was performed as described previously (Jia et al., 2014). For zpr1 immunofluorescence staining, the fish were fixed at 4°C for 3 h with 4% PFA in PBS (pH 7.4), rinsed with PBS (three times, 5 min each), and equilibrated in 30% sucrose in PBS overnight. Then, the samples were transferred into O.C.T. (Tissue-Tek) and sectioned at 10 μm on a cryostat (Leica CM1950). The sections were incubated overnight at 4°C, followed by blocking solution (1% bovine serum albumin, 0.4% Triton X-100 and 8% goat serotonin in PBS) for 40 min and then incubation with zpr-1 antibody (mouse, 1:500; Abcam, Cambridge, MA, USA) for 2 h at room temperature. After washing with PBS (three times, 5 min each), the samples were incubated for 2 h at 4°C with Alexa488-conjugated goat anti-mouse (1:1000 dilution, Invitrogen) in blocking solution, counterstained with propidium iodide (PI), and then mounted with 80% glycerol in PBS for fluorescence microscopy.

### Electron Microscopy

Tissues were processed as described previously (Allwardt et al., 2001). Briefly, zebrafish were fixed in the morning. Whole embryos and larvae were anesthetized and placed into primary fixative for 15 min at 4°C. The primary fixative was prepared fresh daily and consisted of 1% paraformaldehyde, 1.6% glutaraldehyde, and 3% sucrose in 0.06 M phosphate buffer, pH 7.4. The tissue was rinsed and post-fixed in 2% osmium tetroxide in phosphate buffer for 2 h at 4°C. After rinsing, samples were dehydrated in a graded series of ethanol-water mixture and infiltrated with epon/araldite resin. The tissue was cured for 72 h at 60°C. Ultrathin (50 nm) transverse sections of the retina from larvae of both zebrafish genotypes were stained with uranyl acetate and lead citrate. Sections were viewed and photographed with an FEI Tecnai Spirit (120 kV TEM) transmission electron microscope.

### Statistical Analyses

Data are shown as the mean ± SEM. Statistical significance was determined using a two-tailed unpaired Student’s *t*-test or one-way ANOVA or two-way ANOVA with a significance level of *P* < 0.05. A repeated-measure adjustment was employed when appropriate (e.g., daily gene expression profiles). Two-way ANOVA followed by *post hoc* Bonferroni test was applied when needed (e.g., OKR). In particular, we used three independent samples for analysis and three repeated independent measurements for the gene expression experiment, with similar results. For the JTK-CYCLE analysis, each parameter was recorded and analyzed as previously described by R (Hughes et al., 2010). Statistical analysis was performed with GraphPad Prism5 software (San Diego, CA, USA).

## Results

### *per2* Deficiency Affects Zebrafish Larvae Visual Behavior

**Figure 1:**
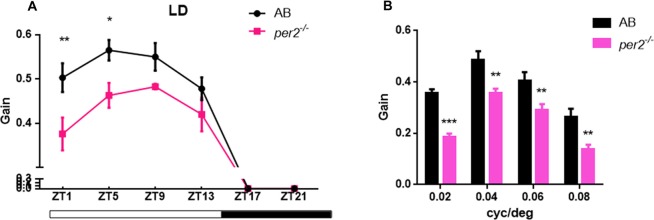
*per2* mutant zebrafish larvae showed optokinetic response (OKR) deficiency. **(A)** OKRs of AB and *per2* mutant during the course of a day under the light/dark (LD) condition (ZT0 light on, ZT14 light off; Contrast = 0.6, *n* = 10; ZT: *F*_(5,85)_ = 124.8, *P* < 0.0001; Genotype: *F*_(1,85)_ = 12.01, *P* = 0.0008). **(B)** OKRs of AB and *per2* mutant under different spatial frequency (SF; contrast = 0.6, *n* = 10; cycle/degree: *F*_(3,40)_ = 30.21, *P* < 0.0001; Genotype: *F*_(1,40)_ = 61.65, *P* < 0.0001). Data from wild type (WT) and *per2* mutant were compared using two-way ANOVA followed by a *post hoc* Bonferroni test. *P < 0.05, **P < 0.01 and ***P < 0.001 compared with the WT. Data represent the mean ± SEM.

**Figure 2:**
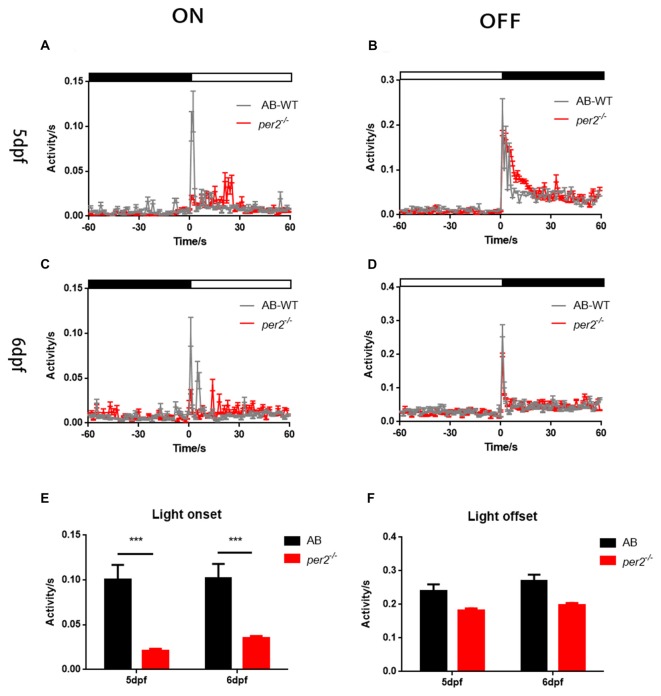
*per2* mutant zebrafish larvae showed visual motor response (VMR) deficiency. **(A–D)** Motor activity in response to light-on **(A,C)** and light-off **(B,D)** at days post-fertilization (dpf; **A,B)** or 6 dpf **(C,D)** of WT (black traces; *n* = 48) and *per2* mutant (red traces; *n* = 48). **(E)** The *per2* mutant larvae displayed an attenuated light-onset compared with that of the WT larvae. **(F)** The *per2* mutant larvae showed no significant of light-offset compared with that of the WT larvae. Data from AB-WT (*n* = 48) and *per2* mutant zebrafish (*n* = 48) were compared using unpaired two-tailed *t*-tests. ***P < 0.001 compared with the WT. Data represent the mean ± SEM.

To determine whether visual responsiveness was altered in the *per2* mutant zebrafish, we recorded the OKR of 5 days post-fertilization (dpf) larvae of the two genotypes at different times of day and night. Compared with the WT, *per2*^−/−^ zebrafish larvae displayed attenuated contrast sensitivity during the day (Figure 1A). Two-way ANOVA showed significant effects of genotype (*F*_(1,85)_ = 12.01, *P* = 0.0008) and ZT (*F*_(5,85)_ = 124.8, *P* < 0.0001). The data also indicated that *per2*^−/−^ zebrafish maintained an OKR circadian rhythm. Another crucial function of the visual system is its ability to distinguish two separate objects at a given angular distance, referred to as visual acuity, and it is influenced by numerous optical and neuronal features of the visual system (Haug et al., 2010). By altering the SF, we found that *per2*^−/−^ mutant larvae showed a significant reduction in gain either at high or low SF (Figure 1B). Two-way ANOVA showed significant effects of genotype (*F*_(1,40)_ = 61.65, *P* < 0.0001). These results suggested that the mutation of clock gene *per2* caused contrast sensitivity and visual acuity deficiency. We conducted another experiment for visual detection using the VMR, which is a primitive startle response displayed by larvae within seconds after the onset and offset of light (Emran et al., 2007; Zhang et al., 2016). For the light-on VMR, the *per2* mutant fish responded weakly at light onset at both 5 dpf (Figure 2A) and 6 dpf (Figure 2C). At 5 dpf, the distance moved variability in 1 s at light-on was 2.41 ± 1.94 mm in WT vs. 0.50 ± 0.27 mm in *per2* mutants (*N* = 48, *P* < 0.01, two-tailed unpaired *t*-test; Figure 2E). At 6 dpf, the distance moved variability in 1 s at light-on was 2.44 ± 1.90 mm in WT vs. 0.84 ± 0.28 mm in *per2* mutants (*N* = 48, *P* < 0.01, two-tailed unpaired *t*-test; Figure 2E). For the light-off VMR, both WT and *per2* mutant fish responded similarly at 5 dpf (Figure 2B) and 6 dpf (Figure 2D). WT and mutant genotypes sharply increased their activity immediately after the cessation of light. At 5 dpf, the distance moved variability in 1 s at light-off was 5.74 ± 2.38 mm in WT vs. 4.38 ± 0.68 mm in *per2* mutants (*N* = 48; Figure 2F). At 6 dpf, the distance moved variability in 1 s at light-off was 6.49 ± 2.13 mm in WT vs. 4.77 ± 0.62 mm in *per2* mutants (*N* = 48; Figure 2F). These results indicated that the *per2* mutant fish readily detected the light-off but responded weakly to the light-on; thus, *per2* is important for the visual behavior response of zebrafish larvae.

### *per2*^−/−^ Zebrafish Larvae Exhibit Abnormal Photoreceptor Ribbon Synapses Compared With Wild-Type Larvae

**Figure 3:**
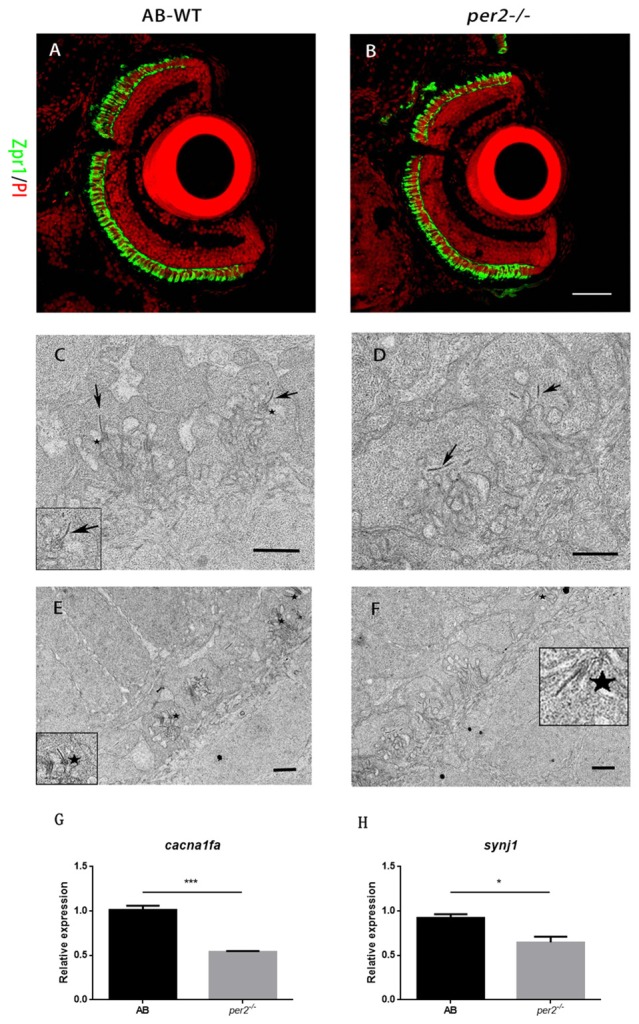
*per2* mutant zebrafish larvae exhibited normal retinal morphology and abnormal photoreceptor ribbon synapses. **(A,B)** AB-WT and *per2*^−/−^ zebrafish larval retinas revealed relatively normal retinal morphology by IHC. Images are LSCM presentations. Larvae at 5 dpf were stained with zpr1 (green) and propidium iodide (PI; red), which label double cones and nuclei (*n* = 6 animals per genotype). Scale bar = 40 μm. **(C–F)** *per2* mutant zebrafish larvae exhibited abnormal ribbon synapses. **(C)** In the AB-WT retina, synaptic ribbons (arrows) are associated with the presynaptic membrane via an arciform density (asterisks). **(D)** In the *per2*^−/−^ retina, synaptic ribbons in most of the pedicles appear to be floating in the cytoplasm and are unassociated with an arciform density and the presynaptic membrane. AB-WT **(E)** had relatively more normal ribbon synapses (asterisks) than those of the *per2* mutant (**F**; *n* = 3 retina per genotype). Scale bar = 1 μm. **(G,H)** Expression of *cacnf1a* and *syjn1* was reduced in mutant compared with WT retinas (5 dpf, *n* = 10 animals per genotype, *P < 0.05 and ***P < 0.001, unpaired two-tailed *t*-tests). Data represent the mean ± SEM.

Retinal structural changes might be relevant to the effect of attenuated visual functions. To determine whether retinal development was defective in *per2*^−/−^ mutant larvae, we studied retinal morphology in *per2*^−/−^ and WT larvae by histologic staining and electron microscopy. The data showed that *per2*^−/−^ mutant embryos had relatively normal numbers of photoreceptors at 5 dpf based on the immunostaining of cryosections with zpr1 (Figures 3A,B), which marks red–green cones (Larison and Bremiller, 1990).

Because *per2* mutant larvae displayed a light-on VMR defect, we investigated whether this visual defect in mutants was the result of abnormal synaptic ribbons (Allwardt et al., 2001). Retinas were studied by electron microscopy at ZT5, which was the time at which the visual function was obviously defected (Figure 1A). Compared with the WT retina, pedicles in the *per2* mutant had the same basic, normal morphology. However, in mutants, the synaptic ribbons were not close to postsynaptic processes and were rarely associated with an arciform density and a presynaptic membrane (Figure 3C); the synaptic ribbons usually appeared to be “floating” in the cytoplasm (Figure 3D). The data also showed the WT larvae (Figure 3E) had more arciform synaptic ribbons than the mutants (Figure 3F). To quantify the pedicle abnormalities of the *per2*^−/−^ mutants, we studied 40 transversely sectioned pedicles from WT and mutant retinas, as previously reported (Allwardt et al., 2001). The results are shown in Table 2. The number of ribbons per pedicle (row 3) was not significantly different between the WT and mutant populations (1.40 ± 0.55 in the WT vs. 1.55 ± 0.7 in mutants, *P* = 0.345; two-tailed unpaired *t*-test). We also conducted an ultrastructural analysis to assess how many ribbons were associated with an arciform density. Of the WT ribbons, 73.2% had an associated arciform density in the plane of section, whereas 38.7% of the mutant ribbons were associated (row 4). These data confirmed that the retinal synapses were affected by the *per2* mutation.

**Table 2:** Ultrastructural characteristics of cone pedicles from *per2* mutants and wild-type (WT) retinas.

	Wild-type	*per2*^−/−^
Pedicles	40	40
Synaptic ribbons	56	62
Ribbons/pedicle	1.40 ± 0.55	1.55 ± 0.7
Ribbons with arciform density	73.2% (41/56)	38.7% (24/62)

A previous study showed that wud (*cacna1fa*) mutants lack synaptic ribbons and that wud is essential for the development of synapses. Synaptojanin 1 (*synj1*) mutants have floating synaptic ribbons (Jia et al., 2014). Thus, we checked the mRNA expression of two genes in the larval retina. We found that the expression of both *cacna1fa* (*P* = 0.007 compared with the WT; Figure 3G) and *synj1* (*P* = 0.022 compared with the WT; Figure 3H) was significantly reduced in mutants. These results suggested that *per2* is required for the expression of *cacna1fa* and *synj1*, which promote synaptic ribbon formation.

### The Expression of Cone Opsin Is Regulated by *per2*

**Figure 4:**
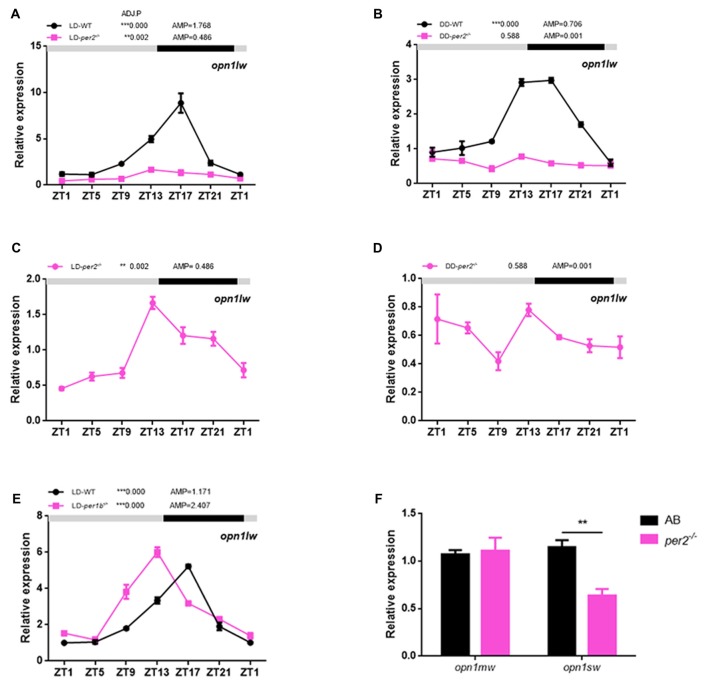
Effects of *per2* mutant on cone opsin expression. **(A,B)** *opn1lw* mRNA was attenuated in *per2*^−/−^ zebrafish larvae under the LD (Genotype: *F*_(1,2)_ = 393.3, *P* = 0.0025) or dark/dark (DD) condition (Genotype: *F*_(1,2)_ = 366.9, *P* = 0.0027). **(C)** Under the LD condition, the circadian rhythms of *opn1lw* mRNA expression remained in *per2*^−/−^ mutant zebrafish larvae (ADJ.P = 0.002, AMP = 0.486). **(D)** Under the DD condition, the circadian rhythms of LC opsin mRNA expression diminished in *per2*^−/−^ zebrafish larvae (ADJ.P = 0.588, AMP = 0.001). **(E)** Under the LD condition, the circadian rhythms of *opn1lw* mRNA expression between *per1b* mutant and WT were different (Genotype: *F*_(1,2)_ = 153.3, *P* = 0.0065). **(F)** The expression of *opn1sw* was reduced in mutants compared with that in the WT at ZT5 (**P < 0.01; unpaired two-tailed *t*-test). No difference in expression was detected for *opn1mw*. Data represent the mean ± SEM. The mRNA expression levels were analyzed by two-way ANOVA repeated measures and the JTK-CYCLE method. ADJ.P for adjusted minimal *p*-values (**P < 0.01, ***P < 0.001), AMP for amplitude.

In zebrafish, the expression of long-wave-sensitive opsin (*opn1lw*) mRNA fluctuates rhythmically in the day and night (Li et al., 2005, 2008). To determine whether there was a significant change in cone opsin expression in *per2*^−/−^ zebrafish, we performed quantitative real-time PCR (qRT-PCR) to detect *opn1lw* mRNA at different times of the day and night while the two genotypes of zebrafish embryos (5 dpf) were kept in either LD or DD conditions. *opn1lw* mRNA is only expressed in the eye (Li et al., 2008). The qRT-PCR analysis revealed a significant effect of genotype during the day in the comparison of mutants with AB-WT in either LD (*F*_(1,2)_ = 393.3, *P* = 0.0025; Figure 4A) or DD (*F*_(1,2)_ = 366.9, *P* = 0.0027; Figure 4B) conditions. In particular, *per2*^−/−^ zebrafish sustained the rhythm of *opn1lw* under the LD condition (ADJ.P = 0.002, AMP = 0.486, JTK-CYCLE; Figure 4C) but were almost rhythm-less under the DD condition (ADJ.P = 0.588, AMP = 0.001; Figure 4D). However, we observed that *per1b*^−/−^ did not dampen the rhythm of *opn1lw* expression compared with that of the WT (*per1b*^−/−^: ADJ.P < 0.0001, AMP = 1.171 vs. WT: ADJ.P < 0.0001, AMP = 2.407; Figure 4E), although *per1b*^−/−^ shifted the *opn1lw* expression in the mutants. Two-way ANOVA showed significant effects of genotype (*F*_(1,2)_ = 153.3, *P* = 0.0065). Additionally, the expression of mRNA of medium-wave-sensitive opsin (*opn1mw*) and short-wave-sensitive opsin (*opn1sw*) did not show rhythm, and that of *opn1sw* was significantly reduced in *per2* mutants (*P* = 0.0026, two-tailed unpaired *t*-test; Figure 4F). These data indicated the important role of *per2* in cone opsin expression.

### The Rhythmic Expression of Clock Genes in *per2* Mutant Has No Obvious Change in Adult Zebrafish Eye

**Figure 5:**
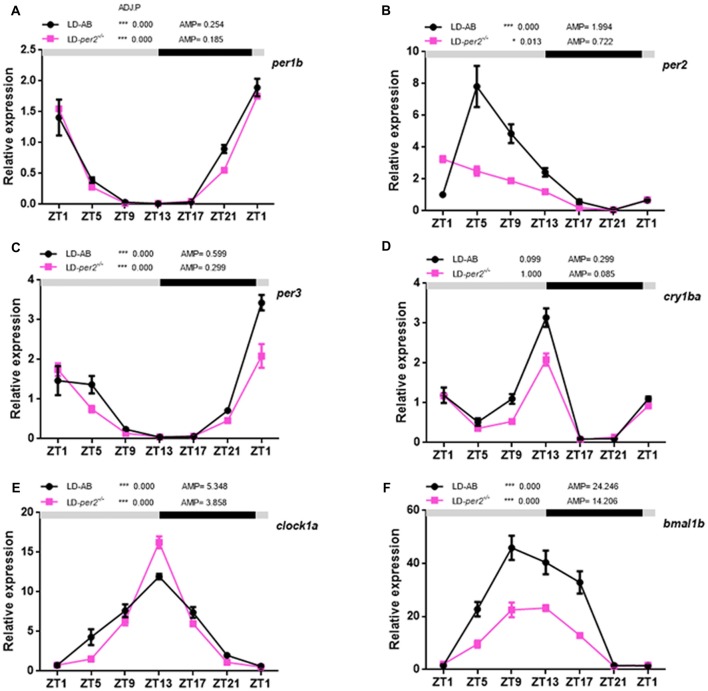
Daily expression of mRNA of key circadian clock genes in *per2* mutant adult zebrafish retinas. **(A–F)** WT and *per2* mutant fish were sacrificed at approximately 3 months of age. Adult zebrafish retinas were dissected out and cleaned, and mRNA was quantified using real-time PCR. Each sample contained at least three female fish and three male fish. qRT-PCR showed the mRNA levels of *per1b, per2, per3, clock1a, cry1ba* and *bmal1b*. Two-way repeated measures ANOVA showed that *per1b* (*F*_(1,2)_ = 2.315, *P* = 0.2675), *per3* (*F*_(1,2)_ = 5.091, *P* = 0.1527), and *clock1a* (*F*_(1,2)_ = 1.154, *P* = 0.3951) were not affected by genotype, whereas *per2* (*F*_(1,2)_ = 24.76, *P* = 0.0381), *cry1ba* (*F*_(1,2)_ = 90.62, *P* = 0.0109) and *bmal1b* (*F*_(1,2)_ = 43.48, *P* = 0.0222) were significantly affected by genotype. The mRNA expression levels were analyzed by two-way ANOVA repeated measures and the JTK-CYCLE method. ADJ.P for adjusted minimal *p*-values (*P < 0.05, ***P < 0.001), AMP for amplitude. Data represent the mean ± SEM.

Zebrafish *per2* is expressed extensively in numerous tissues (Vatine et al., 2009; Ben-Moshe et al., 2014). A previous study demonstrated that *per2* expression is required for the light-induced developmental maturation of the pineal clock (Ziv et al., 2005). An assay with explanted PER2::LUC and *Per1*::LUC mouse retinas showed that *Bmal1*, *Per1*, *Cry1* and *Clock* are each required individually for the retina to express molecular rhythms (Ruan et al., 2012). Here, we investigated the effect of *per2* on a few core circadian oscillation genes in the adult zebrafish retina. Two-way repeated measures ANOVA showed that *per1b* (*F*_(1,2)_ = 2.315, *P* = 0.2675; Figure 5A), *per3* (*F*_(1,2)_ = 5.091; *P* = 0.1527; Figure 5C), and *clock1a* (*F*_(1,2)_ = 1.154, *P* = 0.3951; Figure 5E) were not affected by genotype, whereas *per2* (*F*_(1,2)_ = 24.76, *P* = 0.0381; Figure 5B), *cry1ba* (*F*_(1,2)_ = 90.62, *P* = 0.0109; Figure 5D), and *bmal1b* (*F*_(1,2)_ = 43.48, *P* = 0.0222; Figure 5F) were significantly affected by genotype. In particular, the lack of expression of *per2* in mutant zebrafish is primarily because erroneously synthesized *per2* mRNA is easily degraded. Importantly, the data showed that the *cry1ba* gene obviously damped the rhythm in WT and *per2* mutant zebrafish (WT: ADJ.P = 0.099 vs. *per2*^−/−^: ADJ.P = 1.000; Figure 5D). In fact, this indicates the special expression pattern of the *cry1ba* gene with circadian rhythms. Therefore, *per1b*, *per3*, *cry1ba*, *clock1a* and *bmal1b* had a circadian rhythm in their gene expression. These results indicated that the *per2* plays a positive role in regulation of the expression of *bmal1b* and *cry1ba*.

## Discussion

Clock genes are involved in retinal processing of visual information (Storch et al., 2007; Ait-Hmyed et al., 2013; Mazzotta et al., 2013; Hakkari et al., 2016). In this study, we used *per2* mutant zebrafish larvae, which were generated using the genome-editing tool TALEN (Wang et al., 2015), and found that the gain value of OKR was reduced during the day compared with that of the AB WT. In a previous report, mice lacking Period 1 and Period 2 show no changes in visual responses (Ait-Hmyed et al., 2013). This difference may have occurred because *per2* has a different role in the zebrafish circadian system. Zebrafish *per2* is a light-regulated gene but also has distinct regulatory functions in the different peripheral organs (Vatine et al., 2009; Wang et al., 2015). Additionally, we observed that *per2* mutant zebrafish maintained an OKR circadian rhythm, which might be because mutations in *per2* do not significantly affect the rhythm of the molecular clock or the behavioral rhythm in mutant zebrafish larvae (Wang et al., 2015). We also found that mutations in *per2* did not disrupt the rhythm of the molecular clock of the retina in mutants.

Moreover, *per2* mutant zebrafish larvae showed a light-on VMR defect, which is a phenotype similar to that of *nrc* mutant zebrafish with an ON pathway defect and fewer light-driven ganglion cells (Emran et al., 2007). Our data showed that *per2* mutants exhibited abnormal and decreased arciform ribbon synapses (Figures 3D,F), and these arciform ribbon synapses are mainly formed between ON bipolar and horizontal cells. As mentioned previously, *wud* mutants display abnormal ERGs and cone synaptic ribbon formation is defective (Jia et al., 2014). This finding indicates that a synaptic ribbon defect could account for the attenuated visual function. Additionally, a previous study demonstrated that *per1b* mutant zebrafish eyes exhibit retinal dopaminergic deficiency (Nie et al., 2018), and the expression of genes that play roles in specification, differentiation, and development or maintenance of dopaminergic neurons is significantly downregulated in *per1b* mutants (Huang et al., 2015). However, the question of whether the characteristics of *per2* mutants are showed up in single or double mutations in other clock genes requires further study.

We also examined the possible effects of zebrafish *per2* on the expression levels of cone opsin because the fluctuation of behavioral red cone sensitivity correlates with circadian expression of *opn1lw* during the first 24 h of constant conditions (Li et al., 2005). Ait-Hmyed reported that cone opsin mRNA is reduced in mice lacking Period 1 and Period 2 (Ait-Hmyed et al., 2013). Moreover, the *clock* gene is required to maintain the circadian rhythms of *opn1lw* expression, and the circadian rhythms of *opn1lw* expression are diminished by anti-clock morpholinos in zebrafish embryos (Li et al., 2008). Our results showed that *per2* gene mutants significantly down regulated *opn1lw* expression, with a 3-fold decrease, and diminished the rhythm of *opn1lw* expression under the DD condition. These results suggested that *opn1lw* mRNA is mediated by light and by an endogenous circadian mechanism. According to a previous report, CLOCK may regulate the circadian rhythms of *opn1lw* expression via cAMP in the zebrafish retina (Li et al., 2008). Thus, we hypothesized that PER2 as another clock gene might regulate the circadian rhythms of *opn1lw* expression via cAMP signaling pathways. However, *per3* expression significantly decreased in anti-Clock morpholine embryos (Li et al., 2008), and the *per2* mutant genotype did not significantly affect *clock1a* gene expression in the retina (Figure 5E). Moreover, CLOCK mutation can damp the circadian expression of the clock gene *mPer2* in mouse SCN and liver (Oishi et al., 2005; Vitaterna et al., 2006). Combining the above evidence, we hypothesized that *per2* might be downstream of CLOCK to control the *opn1lw* expression. Further studies will address these questions.

In a previous report, Per1 and Per2 single gene deletion mice showed different responses to photic conditions (Zheng et al., 2001), and *Per2^Brdm 1^* showed a progressive loss of rhythmic behavior. Ait-Hmyed did not examine single Per gene mutant mice, so we do not currently know whether blue cone deficits are in *mper1* mutant or *mper2* mutant strains (Ait-Hmyed et al., 2013). In this study, we found that zebrafish *per1b*^−/−^ and *per2*^−/−^ had different effects in regulating *opn1lw* mRNA expression. Short-wave-sensitive opsin (*opn1sw*) was also down regulated in *per2* mutants. These data suggest that different clock genes have different roles in visual function.

A recent study showed that zebrafish *per2* is important for the circadian clock (Wang et al., 2015). *Per2* mutant zebrafish displayed reduced activities under LD and a 2-h phase delay and a 1.1-h prolonged period under DD conditions (Wang et al., 2015). To determine whether *per2* is essential for the expression of molecular circadian rhythms in the retina, we examined the expression of key clock genes in adult mutant retinas. We found that *bmal1b* was down regulated in *per2* mutants under the LD condition (Figure 4F). In a previous report, *Bmal1* regulated retinal visual processing in the retina, and a b-wave was reduced in *Ret-Bmal1*^−/−^ mice (Storch et al., 2007). Thus, our results indicated that *bmal1b* might also have an important influence on vision in zebrafish.

Our previous study demonstrated that circadian misalignment does not lead to contrast defects during early development in zebrafish larvae (Nie et al., 2018). Under a constant DD conditions, *per2* gene was significantly reduced in zebrafish larvae (Jensen et al., 2012), and zebrafish larvae had normal contrast sensitivity compared with that of the WT larvae (Nie et al., 2018). In this study, *per2* mutant zebrafish larvae displayed reduced vision behavior due to the influences on arciform ribbon synapses, although the rhythm of clock genes was normal in the retina. These data provide evidence that clock genes affect visual function mainly by affecting neurodevelopment and not via circadian misalignment. Finally, this study implicates a major role for *per2* in modulating visual information processing.

## Author Contributions

DH conceived the study, carried out the experiments and helped design them, wrote the manuscript and conceived the figures. MW, WY and YM participated in the design of the studies, data collection. HW, TX, DR and BH helped design the experiments and helped in drafting the manuscript.

## Conflict of Interest Statement

The authors declare that the research was conducted in the absence of any commercial or financial relationships that could be construed as a potential conflict of interest.
